# Specificity of *Phlebotomus papatasi* and *Phlebotomus tobbi* recombinant salivary proteins developed as serological surveillance tool

**DOI:** 10.1186/s13071-026-07479-x

**Published:** 2026-06-04

**Authors:** Helena Přibylová, Metin Pekagirbas, Suha K. Arserim, Kardelen Yetismis, Umut Berberoglu, Unal Altug, Shirly Elbaz, Yaarit Nachum‑Biala, Oscar David Kirstein, Gad Baneth, Yusuf Özbel, Seray Töz, Carla Maia, Petr Volf, Iva Kolářová

**Affiliations:** 1 Department of Parasitology, Faculty of Science, Charles University, Prague, Czechia; 2 Veterinary School, Department of Parasitology, Aydin Adnan Menderes University, Aydin, Türkiye; 3 Vocational School of Health Sciences, Celal Bayar University, Manisa, Türkiye; 4 Department of Parasitology, Faculty of Medicine, Ege University, Izmir, Türkiye; 5 General Directorate of Public Health, Turkish Ministry of Health, Ankara, Türkiye; 6 Ministry of Health Central Laboratories for Public Health, Jerusalem, Israel; 7 School of Veterinary Medicine, The Hebrew University of Jerusalem, Rehovot, Israel; 8 Global Health and Tropical Medicine (GHTM), LA-REAL, Instituto de Higiene e Medicina Tropical (IHMT),, Universidade NOVA de Lisboa (UNL), Lisboa, Portugal

**Keywords:** *Phlebotomus papatasi*, *Phlebotomus tobbi*, Yellow-related protein, Apyrase, D7-related protein, SP32, SP36, SP38, Dog, Mice, Serology, Specificity, ELISA

## Abstract

**Background:**

Sand flies are vectors of various pathogens, primarily *Leishmania* and phleboviruses. The effectiveness of disease control strategies can be assessed using serological tests that measures antibodies against sand fly saliva as a marker of host exposure and a proxy biomarker of sand fly-borne pathogens transmission. Recently, we proposed novel recombinant salivary proteins to evaluate exposure of dogs to *Phlebotomus tobbi* and *P. papatasi*. The aim of this study was to test vector and host specificity of these recombinant proteins.

**Methods:**

Recombinant salivary proteins of *P. papatasi* (PAP-rSP32, PAP-rSP36, and PAP-rSP42) and *P. tobbi* (TOB-rSP10, TOB-rSP38, TOB-rSP56, TOB-rSP60) were tested in an enzyme-linked immunosorbent assay (ELISA) using sera from dogs and mice experimentally bitten by a single sand fly species as well as from naturally exposed dogs.

**Results:**

Among the three *P. papatasi* recombinant proteins tested, rSP36 apyrase exhibited the highest vector species-specificity. It did not cross-react with antibodies from dogs or mice experimentally bitten by *P. perniciosus*, *P. tobbi*, *P. sergenti*, *Sergentomyia schwetzi*, *Lutzomyia longipalpis*, or two mosquito species (*Culex pipiens molestus* and *C. quinquefasciatus*), closely followed by rSP42 yellow-related protein. In contrast, PAP-rSP32 showed host-related species specificity, cross-reacting with murine anti-*P. perniciosus* antibodies, but not with canine ones. Among the four *P. tobbi* recombinants, rSP38 yellow-related protein was the only vector subgenus-specific antigen across all the murine sera tested, followed by rSP10 apyrase and rSP60 D7-related protein, which cross-reacted with anti-*P. papatasi* IgG. The other D7-related protein, rSP56, lacked sand fly-specificity as it cross-reacted with antibodies against *Culex* saliva.

**Conclusions:**

ELISA assays based on PAP-rSP36, PAP-rSP42, and TOB-rSP38 are recommended for large-scale field studies as they exhibited the highest species-specificity. These assays can provide epidemiologically relevant data that complement other surveillance and leishmaniasis control tools.

**Graphical Abstract:**

**Figure:**
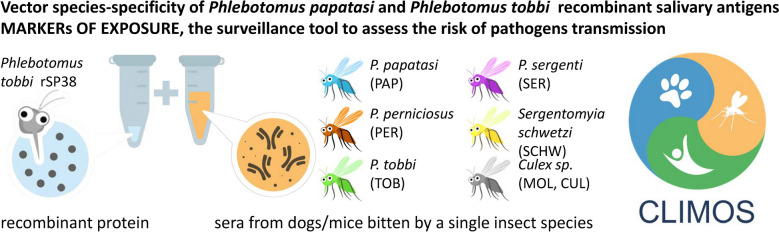

**Supplementary Information:**

The online version contains supplementary material available at 10.1186/s13071-026-07479-x.

## Background

The saliva of sand flies (Diptera: Phlebotominae) is a powerful tool that enables the female fly to overcome the host’s hemostatic reactions and complete the blood meal, thus obtaining nutrients required for egg development. During blood feeding, salivary proteins are injected into the host skin, where they interact with the host immune system. In repeatedly bitten hosts, these proteins elicit a specific antibody response that can be utilized as a marker of exposure to sand flies, and consequently as a risk marker of sand fly-borne pathogens transmission [1]. The use of anti-saliva antibodies as markers of vector exposure is a well-established concept for sand flies [1–4] and has been also demonstrated in other hematophagous arthropods [5], including mosquitoes [6–9], black flies [10, 11], and kissing bugs [12, 13].

Recently, we developed new enzyme-linked immunosorbent assays (ELISAs) for *Phlebotomus papatasi* and *P. tobbi*, in which the salivary gland homogenate was replaced by recombinant salivary proteins as the antigen [3]. This approach made the assays independent of sand fly colony maintenance [14] and manual dissection of salivary glands [15]. The aim of this follow-up study was to further characterize these recombinant proteins in terms of their vector and host specificity.

Vector species specificity is a key aspect of the assays, as it substantially influences the interpretation of results, especially in settings where multiple sympatric sand fly species coexist. Anti-sand fly saliva antibodies are generally species-specific, with only partial cross-reactivity observed among closely related species, typically within the same subgenus [1, 16–18]. Based on the specificity of the recombinant proteins, the assays could be designed to target a specific vector, span closely related vectors transmitting the same *Leishmania* species (e.g., *Le. infantum*, mainly transmitted by sand flies from the subgenus *Larroussius* [19–21]), or be broadly sand fly-specific by using genus-shared salivary antigen or a combination of species-specific proteins.

Host species specificity represents another important parameter affecting the applicability of the recombinant salivary proteins. In our previous study, candidate antigens were selected on the basis of their reactivity with sera from repeatedly bitten dogs [3]. Although the concept of exposure markers can be applied to different host species, the antibody responses are usually host species-specific, since the dominant salivary antigens may differ between host species [22–24]. Consequently, the recombinant proteins originally developed for screening canine sera should be evaluated before application to other host species.

Therefore, in this study, we tested both vector and host species specificity of recombinant salivary proteins using sera from dogs and mice experimentally bitten by a single sand fly species. Sera from naturally exposed dogs were used to indicate the applicability of the recombinant proteins as epidemiological markers of exposure in other epidemic settings, supporting both veterinary and public health surveillance.

## Methods

### Dog sera

Sera from naturally exposed dogs were collected in Türkiye (Manisa) and Israel (Be'er Sheva subdistrict), in areas abundant for *P. papatasi* [25, 26]. Sera from dogs experimentally bitten by *Lutzomyia longipalpis* and *P. perniciosus* were obtained during previous studies as described in Hostomska et al. [27] and Vlkova et al. [28], respectively. Negative control sera originated from the Czechia (a sand fly-free area) or from pre-immune sera collected prior to experimental exposure.

### Mouse sera

Sera were collected from laboratory mice experimentally exposed to bites of either sand flies (*P. papatasi*, *P. perniciosus*, *P. tobbi*, *P. sergenti*, *Sergentomyia schwetzi*) or mosquitoes (*Culex quinquefasciatus*, or *C. pipiens molestus*). Each mouse was repeatedly exposed to approximately 100–150 females of a single insect species at biweekly intervals for about 1 year. Negative control sera were collected from unexposed mice.

### Sand fly salivary gland homogenate

Laboratory colonies of *P. papatasi* and *P. tobbi* were established in 2005 and 2008, respectively, from field-collected sand flies in the Cukurova region, Türkiye. They were maintained under standard conditions in the insectary at the Department of Parasitology, Charles University, Czechia [14, 29]. Salivary glands were dissected from 4- to 6-day-old female sand flies into 0.9% NaCl and stored at −20 °C. Before use, salivary glands were disrupted by three freeze/thaw cycles in liquid nitrogen to obtain the salivary gland homogenate (SGH).

### Recombinant proteins production

Recombinant proteins (TOB-rSP10, TOB-rSP38, TOB-rSP56, TOB-rSP60, PAP-rSP32, PAP-rSP36, and TOB-rSP42) were produced in the *Escherichia coli* expression system at the Core Facility Protein Production (The Centre of Molecular Structure, Institute of Biotechnology, Czech Academy of Sciences) as detailed in [3]. Briefly, codon optimized coding sequence without a signal peptide was cloned into the modified pET-28aN( +)-TEV vector and expressed in the *E. coli* BL21(DE3) cells (New England Biolabs) as His-tagged proteins. After induction, expressed recombinants were collected and purified using PureCube 100 Ni-INDIGO agarose resin (Cube Biotech). Positive fractions were pooled and dialyzed in SnakeSkin™ Dialysis Tubing (Thermo Scientific), followed by a concentration step using VivaSpin 10 kDa-HY (Sartorius). Each protein sample was aliquoted, snap-frozen in liquid nitrogen, and stored at −80 °C.

### ELISA

ELISA was performed as previously described [3]. Briefly, plates (Thermo Scientific #3855) were coated with recombinant protein (0.2 µg/well) or SGH (equivalent to 0.1 gland per well) for 2 h at room temperature (RT), blocked, and incubated with sera diluted 1:300 overnight at 4 °C. The next day, plates were washed and incubated with HRP-conjugated secondary antibody diluted in PBS-Tw as follows: anti-dog IgG 1:40,000 (Bethyl Laboratories, #A40-123P) or anti-mouse IgG 1:5,000 (Bio-Rad, #STAR-120P). The chromogenic reaction was developed in 3,3',5,5'-tetramethylbenzidine substrate solution (Sigma-Aldrich, #T4444). The absorbance was measured at 450 nm. Each plate contained two technical controls: blank (replacing primary and secondary antibody with diluent) and conjugate control (replacing primary antibody with diluent).

### Statistical analysis

**Figure 1:**
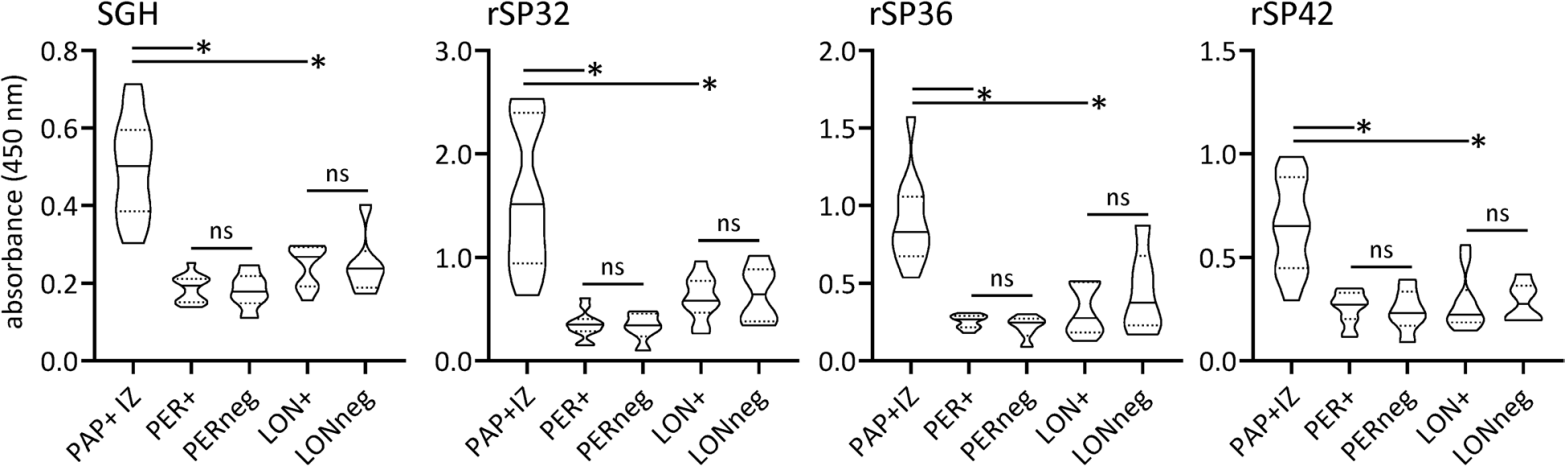
Cross-reactivity of *Phlebotomus papatasi* (PAP) recombinant proteins with sera from dogs exposed to sand fly bites. Salivary gland homogenate (SGH) and recombinant proteins (rSP) were tested in ELISA by using sera from dogs naturally exposed to local sand fly fauna in Israel (IZ, *n* = 12), and sera from dogs experimentally bitten by *P. perniciosus* (PER+, *n* = 12) or *Lutzomyia longipalpis* (LON+, *n* = 10). Preexposed sera were used as negative controls (PERneg, LONneg, *n* = 6 each). The data are presented as a violin plot with the solid line marking the median value and the dotted lines representing Q1 and Q3 quartiles. Asterisks (*) indicate a significant (*P* < 0.05) difference between the PAP+IZ group and PER+/LON+ groups, *ns* not significant (*P* > 0.05)

**Figure 2:**
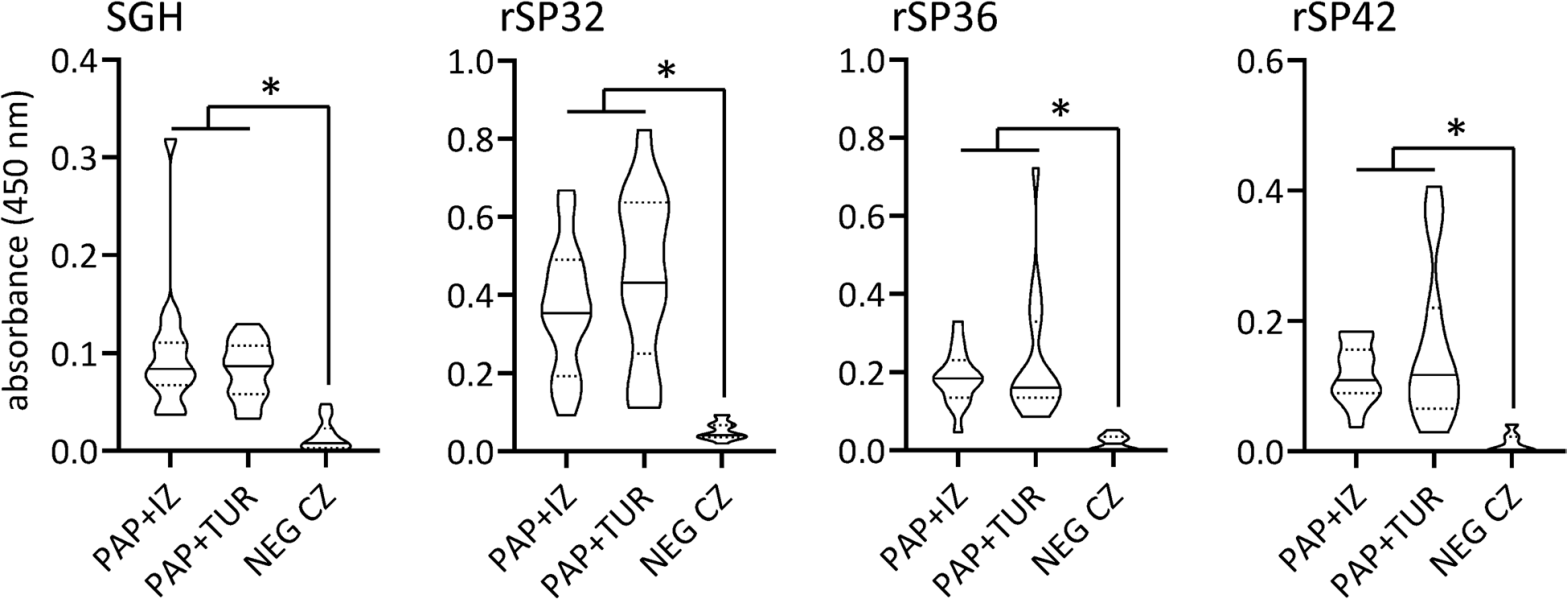
Reactivity of *Phlebotomus papatasi* (PAP) recombinant proteins with sera from dogs naturally exposed to sand fly bites. Salivary gland homogenate (SGH) and recombinant proteins (rSP) were tested in ELISA to detect anti-*P. papatasi* IgG in sera from dogs naturally exposed to local sand fly fauna in Israel (PAP+IZ, *n* = 6) and in Türkiye (PAP+TUR, *n* = 6). Canine sera from Czechia, a sand fly-free area, were used as negative controls (NEG CZ, *n* = 6). The data are presented as a violin plot with the solid line marking the median value and the dotted lines representing Q1 and Q3 quartiles. Asterisks (*) indicate a significant (*P* < 0.05) difference between PAP+ groups compared with NEG control. No significant difference (*P* > 0.05) was observed between the two PAP+ groups for any of the tested antigens

**Figure 3:**
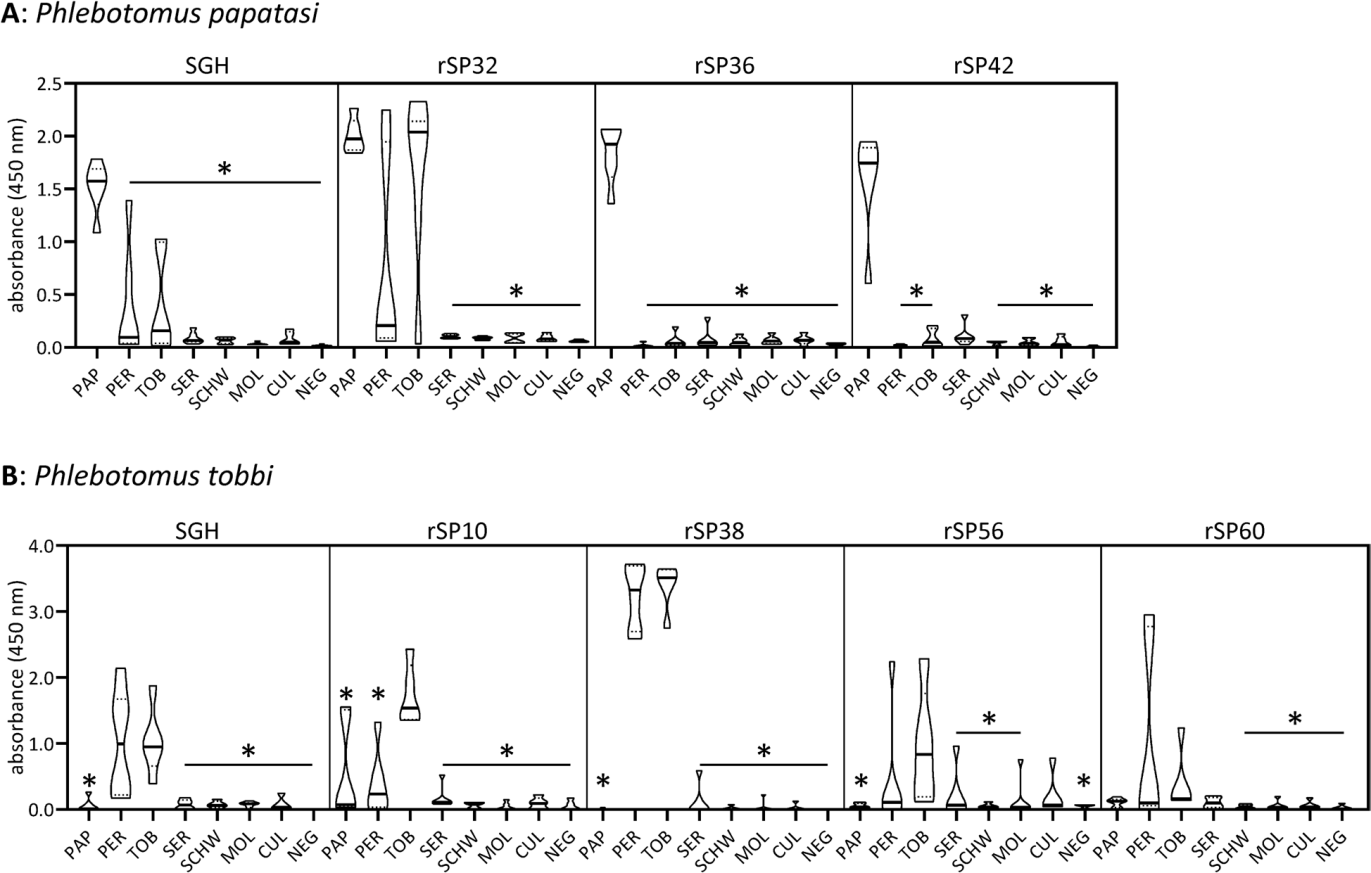
Reactivity of recombinant proteins with sera from mice experimentally bitten by a single insect species. *Phlebotomus papatasi* (**A**) and *P. tobbi* (**B**) salivary gland homogenate (SGH) and recombinant proteins (rSP) were tested in ELISA to detect cross-reacting IgG antibodies in sera from mice repeateadly bitten either by *P. papatasi* (PAP), *P. perniciosus* (PER), *P. tobbi* (TOB), *P. sergenti* (SER), *Sergentomyia schwetzi* (SCHW), *Culex quinquefasciatus* (CUL), or *C. pipiens molestus* (MOL). Non-bitten mice were used as controls (NEG). The data are presented as the violin plot with the solid line marking the median value (*n* = 5–6) and the two dotted lines representing the Q1 and Q3 quartiles. Asterisks (*) indicate significant differences (*P* < 0.05) between PAP and non-PAP sera (**A**) and between TOB and non-TOB sera (**B**)

Statistical analysis was performed using GraphPad Prism v8.0.1. The samples were tested in duplicate, and the average values after conjugate control subtraction were used for downstream analyses. Based on the results of normality test, the difference between groups was evaluated using nonparametric Kruskall–Wallis analysis of variance (ANOVA) with uncorrected Dunn’s multiple comparison (Figs. 1, 2, 3, S1). A *P*-value < 0.05 was considered to be statistically significant.

## Results

### Vector species specificity of *P. papatasi* recombinant proteins in dogs

To evaluate species specificity of *P. papatasi* recombinant antigens as markers of exposure, they were tested with sera from dogs experimentally bitten by a single sand fly species, either *P. perniciosus* or *L. longipalpis*, and with sera from dogs naturally bitten by sand flies in Israel, collected in areas where *P. papatasi* is a dominant sand fly species [26]. All *P. papatasi* antigens followed the same pattern; they reacted strongly with sera from Israeli dogs, while their reactivity with anti-*P. perniciosus* and anti-*L. longipalpis* IgG was significantly lower (*P* < 0.001) and comparable to negative controls (Fig. 1). The presence of anti-*P. perniciosus* IgG in experimentally bitten dogs was confirmed by using *P. perniciosus* rSP03B recombinant protein as a homologous control (Fig. S1).

Moreover, sera from dogs naturally bitten by sand flies in *P. papatasi*-abundant areas in Israel possessed significantly higher anti-*P. papatasi* SGH IgG levels than controls from Czechia, a nonendemic country (*P* < 0.05), and were comparable to levels of anti-*P. papatasi* antibodies in Turkish dogs (*P* > 0.05) (Fig. 2). All three *P. papatasi* recombinant proteins tested exhibited the same pattern (Fig. 2 and S2).

### Vector species specificity of recombinant proteins in mice

Putative cross-reactivity of *P. papatasi* and *P. tobbi* SGH and recombinant salivary proteins has been evaluated with antibodies elicited by salivary proteins from other vector species. Cross-reactivity was tested using a panel of sera from mice repeatedly exposed to one of the following sand fly and mosquito species: *P. papatasi, P. tobbi, P. perniciosus, P. sergenti, Sergentomyia schwetzi, Culex quinquefasciatus*, or *C. pipiens molestus*. Strong reactivity was observed in homologous antigen–antibody combinations (*P. papatasi* antigens with anti-*P. papatasi* IgG and *P. tobbi* antigens with anti-*P. tobbi* IgG). In both cases, antibody levels were significantly higher than those in the corresponding negative controls for each of the tested antigens (Fig. 3). Cross-reactivity was defined as a nonsignificant difference between the heterologous and homologous antibodies, additionally supported by the overlapping range of antibody levels between the two groups.

Among *P. papatasi* antigens, rSP36 and rSP42 were highly specific toward the sera from mice bitten by other tested insect species. On the other hand, strong cross-reactivity with heterologous sera was observed for rSP32 in a combination with sera from mice bitten by either *P. perniciosus* or *P. tobbi*. Despite the significant difference, partial cross-reactivity was also observed between *P. papatasi* SGH and anti-*P. perniciosus* antibodies (Fig. 3A).

Among *P. tobbi* antigens, SGH and rSP38 were the most specific, cross-reacting exclusively with the sera of mice bitten by *P. perniciosus*. Results obtained with rSP10 and rSP60 demonstrated cross-reactivity with sera of mice bitten by *P. papatasi*. Additionally, rSP60 showed cross-reactivity also with anti-*P. sergenti* IgG. In contrast, rSP56 showed the lowest level of species specificity, cross-reacting with sera from mice bitten by *P. perniciosus, P. sergenti*, and the two *Culex* species. (Fig. 3B).

## Discussion

The effectiveness of control strategies for vector-borne diseases can be assessed by measuring antibodies formed against vector saliva as an indicator of host exposure. These measurements, along with pathogen detection in vectors and/or hosts, substantially contribute to our understanding of the epidemiology of these diseases in endemic areas.

Vector species specificity of the recombinant salivary antigen is a critical factor for accurate interpretation of serological assays. However, assessing this species specificity requires well-characterized sera, ideally obtained from hosts experimentally bitten by a single vector species. In this study, we utilized sera from dogs and mice previously subjected to controlled experimental exposures.

All *P. papatasi* antigens (SGH and recombinants) demonstrated a high level of specificity at the genus level (*Phlebotomus*) with no cross-reactivity detected against murine antibodies elicited by *Culex* or *Sergentomyia schwetzi*, nor against canine anti-*L. longipalpis* saliva antibodies. Similar genus-specific reaction was observed in the previous study, when *P. perniciosus* SGH and PER-rSP03B recombinant did not react with antibodies from mice bitten by *Culex pipens molestus, Ixodes ricinus*, or *Ctenocephalides felis* [30].

On the contrary, the cross-reactivity of *P. papatasi* antigens with antibodies against saliva of other *Phlebotomus* species was specific for the host species, vector species, or the method used. Previous studies reported that *P. papatasi* SGH did not react with anti-*P. perniciosus* antibodies from hamsters and humans [18, 31]. Similarly, in our study, none of the *P. papatasi* antigens cross-reacted with canine anti-*P. perniciosus* antibodies. However, *P. papatasi* SGH showed partial cross-reactivity with anti-*P. perniciosus* antibodies from mice. This cross-reactivity was more pronounced and statistically significant in PAP-rSP32, whereas no cross-reactivity was detected in PAP-rSP36 or PAP-rSP42. Similar cross-reactivity level was observed with murine sera against *P. tobbi*, a species closely related to *P. perniciosus* (both from *Larroussius* subgenus) [19, 20, 32].

*Phlebotomus papatasi* occurs sympatrically with *P. perniciosus* and *P. tobbi* in the western and eastern Mediterranean regions, respectively [19, 32]. Therefore, distinguishing among them in serological assays is epidemiologically important, as they transmit different *Leishmania* species (*Le. major* by *P. papatasi* and *Le. infantum* by *Larroussius* species) [19, 20, 33]. *Phlebotomus sergenti*, the vector of *Le. tropica*, is another sand fly species that co-occurs with *P. papatasi* in several endemic foci [19, 20, 33]. Previous studies reported that anti-*P. sergenti* murine antibodies showed partial cross-reactivity with *P. papatasi* SGH in western blot and dot blot assays [34–36]. However, in ELISA-based assays the reactivity was species-specific [34], consistent with our findings using *P. papatasi* recombinant antigens.

This study is the first to evaluate the specificity of *P. tobbi* salivary proteins. In mice, TOB-SGH and the recombinant TOB-rSP38 protein showed highest specificity, cross-reacting only with anti-*P. perniciosus* antibodies. Since both species are closely related, they transmit the same *Leishmania* species, and are allopatric [19, 20, 32, 33]; differentiating between these two species may be of limited epidemiological relevance in many endemic settings. In contrast, other *P. tobbi* recombinant proteins showed lower specificity, with cross-reactivity observed with anti-*P. papatasi* (rSP10 and rSP60) and anti-*P. sergenti* IgG (rSP56 and rSP60). *Phebotomus papatasi* and *P. sergenti* commonly co-occur with *P. tobbi* in some endemic regions, but are vectors of different *Leishmania* species [19, 20, 33], therefore such cross-reactivity may limit the epidemiological utility of these antigens for exposure assignments at the species-level. Finally, as these recombinant proteins did not cross-react with all heterologous anti-sand fly antibodies (rSP10, rSP56, rSP60) and/or could not fully differentiate between anti-sand fly and anti-mosquito antibodies (rSP56), they are not suitable as universal pan-*Phlebotomus* exposure markers either.

It remains to be tested whether the observed specificity of recombinant antigens with murine sera also applies to antibodies elicited in dogs and humans, since the antibody response to sand fly saliva could be host specific [18, 23, 24, 35, 37]. This highlights the importance of validating recombinant antigens across multiple host species before their broad epidemiological implementation. For *P. papatasi*, the interpretation of the results is further limited by the use of positive control sera from naturally bitten Israeli dogs [26], which may contain other antibodies with unknown cross-reactivity. Unfortunately, it was not feasible to perform similar specificity tests for canine sera with *P. tobbi* recombinants due to the lack of sera from dogs solely exposed to this species as a positive control. Future validation would benefit from well-defined dog and human sera obtained from controlled, single-species experimental exposures (e.g., *P. papatasi*, *P. tobbi*, and other locally co-occurring sand flies) to confirm the vector-species specificity of recombinant antigens observed in mice.

Finally, the wide geographic distribution of *P. papatasi* may affect the applicability of recombinant antigens derived from the Turkish population when screening canine sera from geographically distant endemic areas. Although some variation exists in salivary proteins among *P. papatasi* populations [38–40], the species is considered homogeneous [41–43]. In our study, Turkish dog sera showed similar levels of anti-*P. papatasi* antibodies as in sera from Israeli dogs, suggesting that these recombinant antigens could serve as markers of exposure in a wider range of *P. papatasi* distribution. Similarly, *P. perniciosus* rSP03B has been proven as a universal marker of *P. perniciosus* exposure for dogs across European endemic areas [44]. Recently, the PAP-rSP36 was employed in a serological study in two other European countries (Spain, Italy), showing variable levels of antibodies in tested dog sera [45], suggesting that this recombinant protein could serve as a universal marker for *P. papatasi* exposure. Together, these findings support the potential cross-regional applicability of selected recombinant salivary markers, although local validation remains advisable.

## Conclusions

On the basis of our findings, PAP-rSP36 (apyrase) and PAP-rSP42 (yellow-related protein) represent the most reliable recombinant antigens for the species-specific detection of host exposure to *P. papatasi*, while TOB-rSP38 (yellow-related protein) is the best-performing marker of exposure to *P. tobbi*. Overall, these antigens demonstrate high vector-species specificity and therefore provide epidemiologically relevant tools for serological screening to assess exposure, and consequently also the transmission risk of sand fly-borne pathogens in endemic areas.

## Supplementary Information

Additional file 1: **Figure S1.** Reactivity of *Phlebotomus perniciosus* (PER) recombinant protein rSP03B with sera from dogs experimentally bitten by *P. perniciosus* (PER, *n* = 12) or *Lutzomyia longipalpis* (LON, *n* = 10). Pre-exposed sera were used as negative controls (PERneg, LONneg, *n* = 6 each). The data are presented as a violin plot with the solid line marking the median value and the dotted lines representing Q1 and Q3 quartiles. Asterisk (*) indicates a significant (*P* < 0.05) difference between the PER+ group compared with other tested sera. No significant difference (*P* > 0.05) was observed among PERneg, LON+, and LONneg groups for any of the tested combinations. **Figure S2.** ROC curve analysis of the *Phlebotomus papatasi* SGH and recombinant proteins with the same sera as in Fig. 2. The two sets of sera from dogs naturally exposed to sand flies in Israel and Türkiye were combined (*n* = 30) and plotted against canine sera from Czechia (*n* = 15), as a control. Antigens used are indicated in each graph.

## Data Availability

Data supporting the main conclusions of this study are included in the manuscript.

## Abbreviations

ANOVA: Analysis of variance
*C*.: *Culex*
CUL: *Culex quinquefasciatus*
CZ: Czechia
ELISA: Enzyme-linked immunosorbent assay
HRP: Horseradish peroxidase
IgG: Immunoglobulin G
IZ: Izrael
*L*.: *Lutzomyia*
*Le*.: *Leishmania*
LON: *Lutzomyia longipalpis*
MOL: *Culex pipiens molestus*
NEG: Negative
*P*.: *Phlebotomus*
PAP: *Phlebotomus* *papatasi*
PER: *Phlebotomus* *perniciosus*
rSP: Recombinant salivary protein
ROC: Receiver operating characteristic
RT: Room temperature
SCHW: *Sergentomyia* *schwetzi*
SER: *Phlebotomus* *sergenti*
SGH: Salivary gland homogenate
TOB: *Phlebotomus* *tobbi*
TUR: Türkiye
