# Supplementary material for: Specificity of Phlebotomus papatasi and Phlebotomus tobbi recombinant salivary proteins developed as serological surveillance tool
Source: Parasit Vectors. 2026 Jun 4;19:334. doi: 10.1186/s13071-026-07479-x (PMC13474924; doi:10.1186/s13071-026-07479-x)
Supplement: Supplementary file 1 [file 13071_2026_7479_MOESM1_ESM.docx]

**Specificity of *Phlebotomus papatasi* and *P. tobbi* recombinant salivary proteins developed as serological surveillance tool**

Helena Přibylová1^#^, Metin Pekagirbas2, Suha K. Arserim3, Kardelen Yetismis4, Umut Berberoglu5, Unal Altug5, Shirly Elbaz6, Yaarit Nachum‑Biala7, Oscar David Kirstein6, Gad Baneth7, Yusuf Özbel4, Seray Töz4, Carla Maia8, Petr Volf1, Iva Kolářová1^#^*

^1^ Department of Parasitology, Faculty of Science, Charles University, Prague, Czech Republic

^2^ Veterinary School, Department of Parasitology, Aydin Adnan Menderes University, Aydin, Türkiye

^3^ Vocational School of Health Sciences, Celal Bayar University, Manisa, Türkiye

^4^ Department of Parasitology, Faculty of Medicine, Ege University, Izmir, Türkiye

^5^ General Directorate of Public Health, Turkish Ministry of Health, Ankara, Türkiye

^6^ Ministry of Health Central Laboratories for Public Health, Jerusalem, Israel

^7^ School of Veterinary Medicine, The Hebrew University of Jerusalem, Rehovot, Israel

^8^ Global Health and Tropical Medicine (GHTM), LA-REAL, Instituto de Higiene e Medicina Tropical (IHMT), Universidade NOVA de Lisboa, Lisboa, Portugal

* Correspondence: [iva.kolarova@natur.cuni.cz](mailto:iva.kolarova@natur.cuni.cz)

^#^ HP and IK share the position of the first author

**Supplementary information**

**Figure S1**. Reactivity of *Phlebotomus perniciosus* (PER) recombinant protein rSP03B with sera from dogs experimentally bitten by *P. perniciosus* (PER, n = 12) or *Lutzomyia longipalpis* (LON, n = 10). Pre-exposed sera were used as negative controls (PERneg, LONneg, n = 6 each). The data are presented as a violin plot with the solid line marking the median value and the dotted lines representing Q1 and Q3 quartiles. Asterisk (*) indicates a significant (p < 0.05) difference between the PER+ group compared to other tested sera. No significant difference (p > 0.05) was observed among PERneg, LON+, and LONneg groups for any of the tested combinations.

**Figure S2.** ROC curve analysis of the *Phlebotomus papatasi* SGH and recombinant proteins with the same sera as in Figure 2. The two sets of sera from dogs naturally exposed to sand flies in Israel and Türkiye were combined (n = 30) and plotted against canine sera from the Czech Republic (n = 15), as a control. Antigens used are indicated in each graph.
